# Providing support to family caregivers through a dementia care conference in the Pacific island of Guam

**DOI:** 10.1002/alz70858_102540

**Published:** 2025-12-25

**Authors:** Iain K. B. Twaddle, Camille Minerva Maestrecampo, Maree Josephine Saloma, Nikolas Jude Gutierrez

**Affiliations:** ^1^ University of Guam, Mangilao, Guam, Guam

## Abstract

In 2020, an online support group for family caregivers of persons with dementia was established in the Pacific island of Guam. Initially, the support group was created to help family caregivers overcome the isolation they faced during the COVID‐19 pandemic. Caregivers met weekly online to share their experiences, participate in virtual consultations with dementia care specialists, and receive dementia care training. After four years, an in‐person conference was organized to bring together support group participants with the goal of strengthening the sense of community that had been established online. The conference incorporated a holistic approach to dementia care with a diverse range of speakers, including healthcare professionals, community agencies serving older adult populations, and family caregivers. It was a full‐day event held in a hotel ballroom to honor family caregivers and the important work they do. Attendance was free of charge with breakfast and lunch included. The conference had seven components: (1) a keynote address titled, “The Journey of Caregiving;” (2) a neurologist's presentation on Alzheimer's disease and other dementias; (3) a neuropsychologist's presentation on aging and dementia; (4) an art therapy session using clay, paint, and mandalas; (5) a meditation and chair yoga session; (6) personal stories shared by family caregivers highlighting challenges, successes, and insights from their caregiving journey; and (7) presentations by community based organizations on resources for older adult populations. Throughout the conference, family caregivers were given the opportunity to connect with one another, meet dementia care specialists, and become part of a community dedicated to the care of older adults. The conference was well attended with over 200 participants including family caregivers, persons with dementia, healthcare professionals, and university students. Participants gave overwhelmingly positive feedback and requested that the conference become an annual event. In conclusion, specialized conferences for family caregivers of persons with dementia can serve as an effective way to provide valuable information on dementia and dementia care support services, to offer therapeutic interventions designed for caregivers and older adults, to create a space for sharing caregiving stories, and to strengthen ties within the dementia care community.

